# Arsenic trioxide attenuates STAT-3 activity and epithelial-mesenchymal transition through induction of SHP-1 in gastric cancer cells

**DOI:** 10.1186/s12885-018-4071-9

**Published:** 2018-02-06

**Authors:** Sung Ho Kim, Hyo Soon Yoo, Moon Kyung Joo, Taehyun Kim, Jong-Jae Park, Beom Jae Lee, Hoon Jai Chun, Sang Woo Lee, Young-Tae Bak

**Affiliations:** 1 Division of Gastroenterology, Department of Internal Medicine, Korea University College of Medicine Guro Hospital, 148, Gurodong-ro, Guro-gu, Seoul, 152-703 Republic of Korea; 2 Division of Gastroenterology, Department of Internal Medicine, Korea University College of Medicine Anam Hospital, 73, Inchon-ro, Seongbuk-gu, Seoul, 136-705 Republic of Korea; 3 Division of Gastroenterology, Department of Internal Medicine, Korea University College of Medicine Ansan Hospital, 123, Jeokgeum-ro, Danwon-gu, Ansan-si, Gyeonggi-do 425-707 Republic of Korea

**Keywords:** Arsenic trioxide, Epithelial-mesenchymal transition, SH2-containing protein tyrosine phosphatase 1, Signal transducer and activator of transcription 3

## Abstract

**Background:**

We investigated the effect of arsenic trioxide (ATO) for inhibition of signal transducer and activator of transcription 3 (STAT3) and epithelial-mesenchymal transition (EMT) in gastric cancer cells, and the role of SH2 domain-containing phosphatase-1 (SHP-1) during this process.

**Methods:**

We used AGS cells, which showed minimal SHP-1 expression and constitutive STAT3 expression. After treatment of ATO, cellular migration and invasion were assessed by using wound closure assay, Matrigel invasion assay and 3-D culture invasion assay. To validate the role of SHP-1, pervanadate, a pharmacologic phosphatase inhibitor, and SHP-1 siRNA were used. Xenograft tumors were produced, and ATO or pervanadate were administered via intraperitoneal (IP) route.

**Results:**

Treatment of ATO 5 and 10 μM significantly decreased cellular migration and invasion in a dose-dependent manner. Western blot showed that ATO upregulated SHP-1 expression and downregulated STAT3 expression, and immunofluorescence showed upregulation with E-cadherin (epithelial marker) and downregulation of Snail1 (mesenchymal marker) expression by ATO treatment. Anti-migration and invasion effect and modulation of SHP-1/STAT3 axis by ATO were attenuated by pervanadate or SHP-1 siRNA. IP injection of ATO significantly decreased the xenograft tumor volume and upregulated SHP-1 expression, which were attenuated by co-IP injection of pervanadate.

**Conclusion:**

Our data suggest that ATO inhibits STAT3 activity and EMT process by upregulation of SHP-1 in gastric cancer cells.

## Background

Arsenic trioxide (ATO) is a chemotherapeutic agent with clinical effects which have been widely accepted in hematopoetic malignancy. In gastric cancer cells, ATO was reported to inhibit cellular proliferation and induce cell cycle arrest via modulation of phosphatidylinositol 3-kinase/Akt (PI3K/Akt) and p53 signaling [1]. The pro-apoptotic effect of ATO in gastric cancer cells is also suggested by inhibition of Akt and mTOR signaling [2]. A recent study showed that ATO may inhibit the signal transducer and activator of transcription 3 (STAT3) activation in alpha-fetoprotein-producing gastric cancer cells, and thereby induce apoptosis [3]. Indeed, STAT3 plays a pivotal role in gastric carcinogenesis and progression, and induces epithelial-mesenchymal transition (EMT) by upregulation of mesenchymal transcription factors such as Snail1 in gastric cancer cells [4]. Thus, inhibition of STAT3 activation is considered a key point of attack to prevent the formation and invasion of gastric cancer. However, direct inhibition of STAT3 may be somewhat ineffective because its large surface area leads to biologically unstable STAT3 inhibitors [5]. Thus, indirect and detour inhibition of STAT3 including dephosphorylation of STAT3 and Janus kinase 2 (JAK2), the upstream internal tyrosine kinase of STAT3, might be a reasonable option.

SH2-containing protein tyrosine phosphatase 1 (SHP-1) is a non-receptor protein tyrosine phosphatase, which is encoded by *PTPN6* on human chromosome 12p13 [6]. As the name implies, SHP-1 inhibits various tyrosine kinases by dephosphorylation, and the JAK2/STAT3 axis is a good example. The anti-STAT3 activity of SHP-1 has been vigorously studied in hematopoietic malignancy [7], however, its role in gastrointestinal solid tumors is not well understood. We previously reported that the protein and messenger RNA (mRNA) expression of SHP-1 is negative or minimal in various gastric cancer cell lines, which are mainly governed by CpG island promoter hypermethylation. Reinforced SHP-1 expression in gastric cancer cells effectively inhibits STAT3 activity and its target genes such as cyclin D1, matrix metalloproteinases-9 (MMP-9), vascular endothelial growth factor-1 (VEGF-1), and survivin [8]. Here, we report on the anti-EMT effect of ATO in gastric cancer cells by demonstrating dephosphorylation of JAK2/STAT3 and modulation of EMT markers including Snail1 and E-cadherin by ATO, and suggest that SHP-1 might be an important mediator for inactivation of the JAK2/STAT3 signaling pathway.

## Methods

### Reagents and cell line

ATO (purity > 99.5%) was purchased from Sigma-Aldrich (St. Louis, MO, U.S.A.), dissolved in NaOH to a concentration of 100 mmol/L and stored at − 20 °C. It was diluted immediately before use to the desired concentrations. The human gastric cancer cell line (AGS) was obtained from Korean Cell Line Bank (Seoul National University, Seoul, Korea), and cultured in RPMI (Gibco, Carlsbad, CA, U.S.A.) supplemented with 10% heat-inactivated FBS (Gibco) and penicillin/streptomycin (1.0%, Gibco-BRL). Cells were incubated at 37 °C in a humidified atmosphere of 5% CO_2_.

### Water-soluble tetrazolium salt-1 (WST-1) cell proliferation assay

To quantify the inhibitory effect of ATO on cellular proliferation, we used a commercial WST-1 assay kit (EZ-CYTOX, Dogen, Seoul, Korea) according to the manufacturer’s instructions [9]. Briefly, 1 × 10^4^ of AGS cells per well were cultured in 96 wells at 37 °C for 24 h, and treated with 5 or 10 μM ATO for 24, 48 and 72 h. Untreated cells, were cultured for the same time period as controls. After ATO treatment, 10 μL of WST was added to each well for an additional 4 h, and absorbance at 450 nm was measured by an ELISA reader (Epoch, BioTek Instruments, Seoul, Korea). All the experiments were performed in triplicate.

### Wound closure assay

After treatment with ATO for 48 h, with or without pervanadate, cells were equally seeded on a 6-well plate chamber. After attachment, a monolayer wound was made by scratching a 200-μL pipette tip along the bottom of the chamber. The media was changed to remove floating debris, and the vertical distance between both sides of the wound, in at least three distinct randomly selected areas, was measured at 24 and 48 h after wound injury using software.

### Matrigel invasion assay

Following the 48 h treatment with ATO, with or without pervanadate, 4 × 10^4^ cells/well were placed in 24-well Matrigel Invasion Chambers (BD Biosciences, Franklin Lakes, NJ, USA) with 2% FBS medium, and 10% FBS was added to the lower wells. After 24 h of incubation, filter membranes were stained with crystal violet, and the number of positive membrane pores was counted in at least five distinct randomly selected areas using 20× magnification.

### 3-D culture spheroid cell invasion assay

A 96 well 3-D spheroid BME cell invasion assay kit was purchased from Trevigen (Gaithersburg, MD, USA), and the assay was performed following the manufacturer’s instructions. Briefly, AGS cells were treated with ATO, with or without pervanadate, for the indicated time; 2 × 10^3^ cells were resuspended in spheroid formation extracellular matrix, and added to each well on the 96-well spheroid formation plate. After 3 days, invasion matrix and medium containing invasion modulating compounds were added, and the images of spheroid in each well were taken by using 4× phase-contrast microscope.

### Western blot analysis

A rabbit polyclonal IgG antibody against human SHP-1 (sc-287), human β-actin (sc-47,778), and a mouse monoclonal IgG antibody against Snail1 (sc-10,433) was purchased from Santa Cruz Biotechnology, Inc. (Santa Cruz, CA, USA). Mouse monoclonal IgG antibodies against human STAT3 (#9139) and p-STAT3 (Tyr705, #4113), and rabbit polyclonal antibodies against human JAK2 (#3230), p-JAK2 (Tyr1007/1008, #3771) and E-cadherin (#3195) were purchased from Cell Signaling Technology (Beverly, MA, U.S.A.). A total of 80–100 μg of whole cell lysate protein was extracted using CelLytic M (C2978; Sigma-Aldrich, St. Louis, MO, USA) with Complete Mini protease inhibitor cocktail (Roche Diagnostics GmbH, Mannheim, Germany). Primary antibodies were diluted at a ratio of 1:1000 in blocking buffer (Tris-buffered saline with Tween-20; Biosesang, Gyeonggi, Korea) containing 5% skim milk (Difco; Becton-Dickinson and Co., Sparks, MD, USA). Probed membranes were incubated for 12 h at 4 °C. The membranes were incubated with goat anti-mouse or anti-rabbit IgG as a secondary antibody for 1 h at room temperature. The protein bands were visualized by exposing the membrane to enhanced chemiluminescence (Perkin-Elmer, Waltham, MA, USA) for 1 min.

### Immunocytochemistry

AGS cells were treated with 10 μM ATO for 24 h and plated on a glass slide, air-dried for 15 min at room temperature and fixed with 3.7% formaldehyde. After washing, slides were incubated with a mouse monoclonal antihuman Snail1 IgG antibody or a rabbit polyclonal antihuman E-cadherin IgG antibody with 1/100 dilution during overnight. And then, slides were washed and incubated with goat antimouse IgG (red, #A-11004) or goat antirabbit IgG (green, #A-11008) for 1 h with 1/100 dilution. Secondary antibodies were purchased from Invitrogen (Waltham, MA, USA). Couterstaining was performed by using 4′,6-diamidino-2-phenylindole (DAPI, blue) (Vectashield, Vector Laboratories, Burlingame, CA, USA) for 5 min. Cells were observed by using a confocal microscope (LSM 700, Carl Zeiss, Oberkochen, Germany) and pictures were captured with digital camera (Carl Zeiss).

### Transfection

For transient small interfering RNA (siRNA) transfection, SHP-1 (sc-29,478) and control siRNA (sc-37,007) were purchased from Santa Cruz. For transfection, cells were cultured for 24 h until they reached 70%–80% confluence and then transfected with 2 μg of plasmid using Lipofectamine 2000 (Invitrogen Life Technologies) according to the manufacturer’s instructions. Cells were collected at 24 h after transfection for further functional analyses.

### Xenograft tumor in nude mice

Six-week-old male Balb/C athymic nude mice (nu/nu) were purchased from Orient Bio (Seongnam, Gyeonggi, Korea). Mice were housed and maintained under pathogen free conditions. Xenograft tumors were produced using a previously described protocol [10]. Briefly, 2 × 10^6^ AGS cells were mixed with Matrigel and injected subcutaneously in both shoulders of each mouse. Two weeks after the AGS cell injection, mice were given intraperitoneal injections of 2 mg/kg of ATO, with or without a once a week addition of 10 mg/kg of pervanadate, 3 times a week for 6 weeks. Intraperitoneal injections of normal saline were used with the same injection schedule in the control group. Tumor size was measured every 3–4 days using a slide caliper. Tumor volume was calculated by the formula 0.44 × A × B^2^ (A = longer diameter, B = perpendicular diameter of A). This in vivo experiment was approved by the Ethic Committee of Korea University Laboratory Animal Research Center.

### Immunohistochemistry

After extraction of xenograft tumors from nude mice, immunohistochemistry (IHC) was performed as previously described [11]. In brief, samples were fixed in 10% formalin, and paraffin embedded. 4-μm sections were adhered to slides, dried overnight, and then deparaffinized overnight and rehydrated. The staining against SHP-1 was performed with anti-SHP-1 antibody, which is the same one used in Western blotting. Slides were incubated with anti-SHP-1 antibody for 30 min at room temperature, followed by an avidin-biotin peroxidase complex. Images were captured by using a fully motorized microscope with a high resolution digital camera.

### Statistical analysis

The SPSS software ver. 19.0 (SPSS, Inc., Chicago, IL, USA) was used for all analyses. Data are presented as median ± standard deviation. A student t test was performed for continuous data, and a *p*-value less than 0.05 was considered as statistical significant.

## Results

### ATO induces SHP-1 expression and attenuates p-JAK2/p-STAT3 to inhibit EMT in gastric cancer cells

**Fig. 1:**
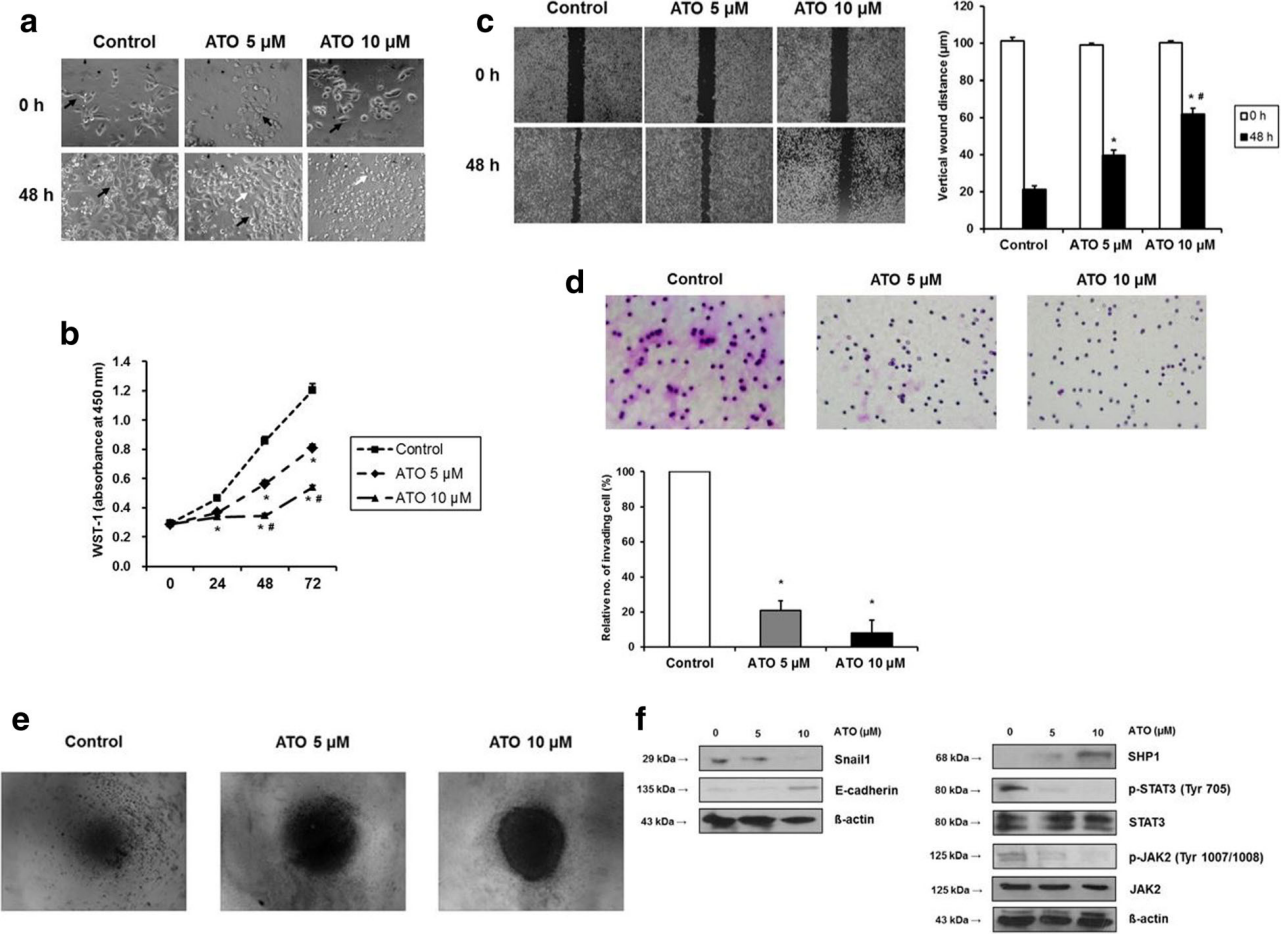
Anti-EMT effects of ATO on AGS cells by induction of SHP-1 and dephosphorylation of STAT3. **a.** Phase contrast microscopy. All images were obtained at a magnification of × 100. **b**. WST-1 cell proliferation assay. All experiments were performed in triplicate. ^*^P < 0.05, compared with control; ^#^P < 0.05, compared with ATO 5 μM (*n* = 3). **c**. Wound closure assay. Left panel; representative images of wound closure. Right panel; analysis of vertical wound distance. Data are presented as mean ± standard deviation. All experiments were performed in triplicate. ^*^P < 0.05, compared with control; ^#^*P* < 0.05, compared with ATO 5 μM (n = 3). **d**. Matrigel invasion assay. Left panel; representative images of Matrigel invasion assay. Right panel; analysis of invading cells. The number of positive invading cells was counted under × 20 magnification. Data are presented as mean ± standard deviation. Cell counting was performed in at least 5 randomly selected separate areas. ^*^P < 0.05, compared with control (*n* = 5). **e**. 3-D culture spheroid cell invasion assay. Images were taken 7 days after resuspension of AGS cells in spheroid formation extracellular matrix, and adding invasion matrix and medium containing invasion modulating compounds. **f**. Western blotting. Whole cell lysate protein was extracted after 48 h treatment with ATO. β-actin was used as an internal loading control

The effect of 48 h treatment with 5 or 10 μM ATO on the morphology of AGS cells was examined by phase contrast microscopy. AGS cells showed a fibroblast-like mesenchymal cell phenotype at baseline, while an epitheloid-like epithelial cell phenotype appeared after treatment with ATO. The epithelial cell phenotype was less commonly seen in the control group (Fig. 1a). The WST-1 assay demonstrated that treatment of AGS cells with 5 and 10 μM of ATO significantly inhibited cellular proliferation at 24, 48 and 72 h (Fig. 1b). The wound closure assay showed that 48 h treatment of AGS cells with 5 and 10 μM ATO significantly increased the vertical wound distance, indicating an anti-migration effect (Fig. 1c). The Matrigel invasion assay showed that the relative number of invading cells was significantly decreased by treatment of AGS cells with 5 and 10 μM of ATO (Fig. 1d). To visually confirm the difference in AGS cell invasion, we performed a 3-D spheroid cell invasion assay after treatment with 5 and 10 μM of ATO for 48 h. Untreated AGS cells protruded out of the spheroid into the surrounding matrix. However, treatment with 5 and 10 μM of ATO dramatically abolished this protrusion into the matrix (Fig. 1e). Western blot showed that 48 h treatment with 5 and 10 μM ATO induced SHP-1 expression and downregulated p-JAK2/p-STAT3 levels in a dose-dependent manner. This in turn downregulated expression of Snail1, a target gene of STAT3 and a marker of mesenchymal transition in gastric cancer cells, and upregulated expression of E-cadherin, a target gene of Snail1 which directly represses E-cadherin expression in epithelial cells [12] (Fig. 1f). Taken together, our data suggest that treatment of AGS cells with ATO significantly inhibited EMT, and that downregulation of p-JAK2/p-STAT3 may play pivotal roles in the anti-EMT effects of ATO.

**Fig. 2:**
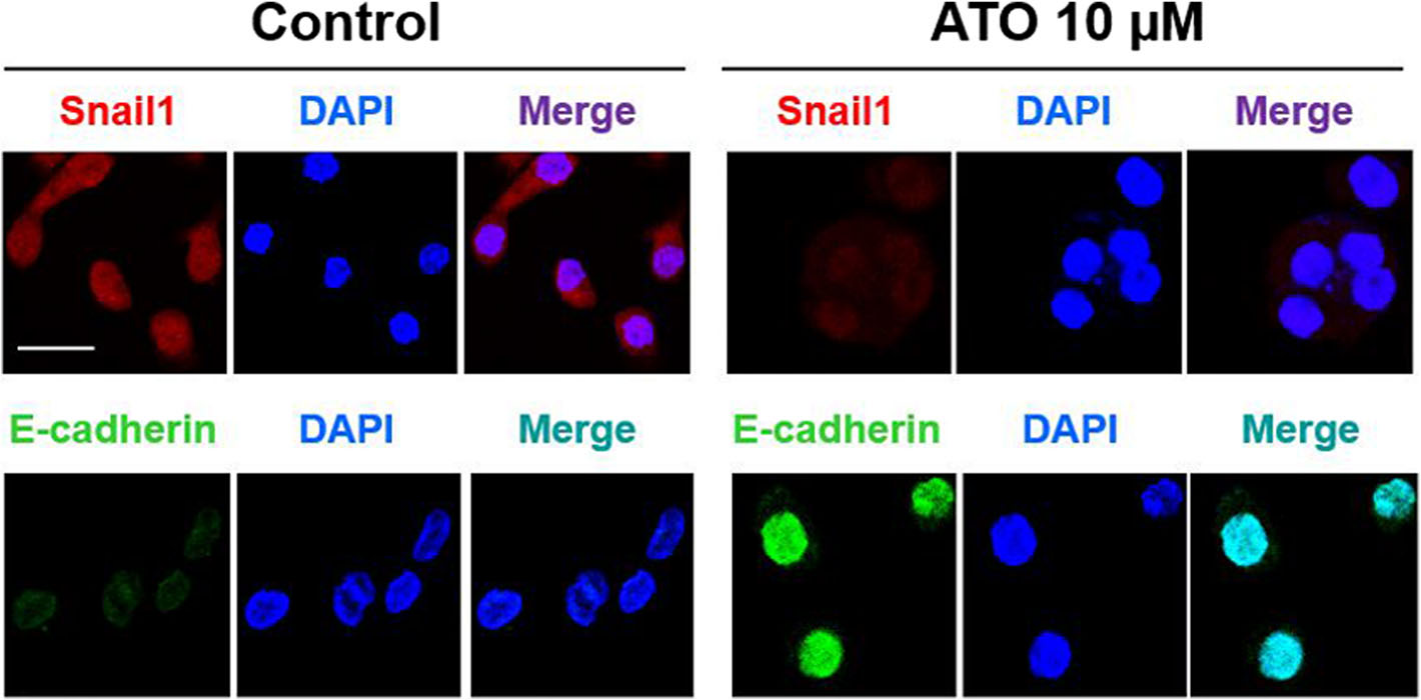
Effects of ATO on AGS cells on cellular phenotype and expression of EMT markers. AGS cells were stained with Snail1 stain (red) or E-cadherin stain (green), 4′,6-diamidino-2-phenylindole (DAPI) stain (blue), and after merging respectively. Treatment of ATO 10 μM to AGS cells showed epithelial phenotype with upregulation with E-cadherin and downregulation of Snail1 expression compared with vehicle-treated AGS cells. Note that white bar indicates 20 μm

To visualize the mesenchymal or epithelial phenotype of AGS cells by staining with EMT markers, we performed immunocytochemistry after treatment of 10 μM ATO for 24 h, which showed round morphology with upregulation of E-cadherin and downregulation of Snail1 expression. On the contrary, vehicle-treated AGS cells showed mesenchymal phenotype with elongated or angulated morphology and downregulation of E-cadherin and upregulation of Snail1 expression (Fig. 2).

### Anti-EMT effect and inhibition of JAK2/STAT3 activity of ATO is attenuated by phosphatase inhibitor

**Fig. 3:**
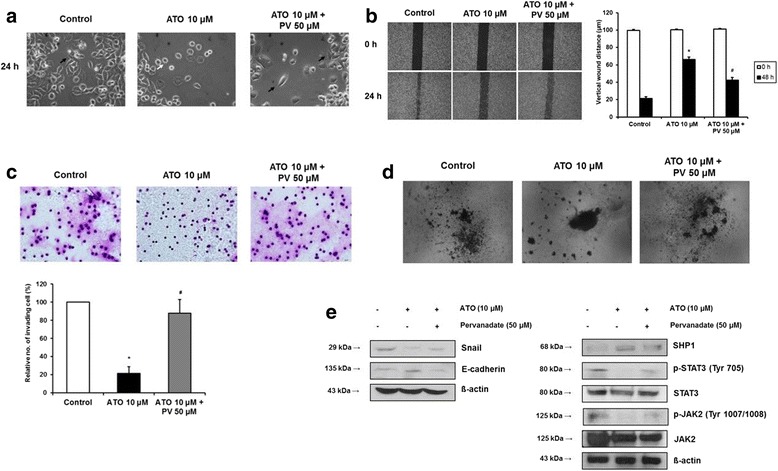
Effects of ATO on AGS cells were reversed by phosphatase inhibitor. **a**. Phase contrast microscopy. All images were obtained at a magnification of × 100. **b**. Wound closure assay. Left panel; representative images of wound closure. Right panel; analysis of vertical wound distance. Data are presented as mean ± standard deviation. All experiments were performed in triplicate. ^*^P < 0.05, compared with control; ^#^P < 0.05, compared with ATO 10 μM (*n* = 3). **c**. Matrigel invasion assay. Left panel; representative images of Matrigel invasion assay. Right panel; analysis of invading cells. The number of positive invading cells was counted under × 20 magnification. Data are presented as mean ± standard deviation. Cell counting was performed in at least 5 randomly selected separate areas. ^*^P < 0.05, compared with control; ^#^P < 0.05, compared with ATO 10 μM (*n* = 5). **d**. 3-D culture spheroid cell invasion assay. Images were taken 7 days after resuspension of AGS cells in spheroid formation extracellular matrix, and adding invasion matrix and medium containing invasion modulating compounds. **e**. Western blotting. Whole cell lysate protein was extracted after 24 h ATO treatment with or without pretreatment with 50 μM pervanadate for 1 h. β-actin was used as an internal loading control

Pervanadate, a pharmacologic phosphatase inhibitor, was used previously to validate the inhibitory effect of SHP-1 in STAT3-activated cancer [13, 14] In this study, pervanadate was used to investigate the role of SHP-1 in the dephosphorylation of JAK2/STAT3 and the anti-EMT effect of ATO. The 24 h treatment of AGS cells with 10 μM ATO changed AGS cell morphology from fibroblast-like mesenchymal phenotype to round, epithelioid phenotype. However, pretreatment with 50 μM pervanadate for 1 h led to the reappearance of mesenchymal-like cells (Fig. 3a). Wound closure assay showed that 24 h treatment of AGS cells with 10 μM ATO significantly increased the vertical wound distance, and that this was reversed by pretreatment with 50 μM pervanadate (Fig. 3b).The Materigel invasion assay also showed a pervanadate reversal of ATO effects in that the ATO-induced decrease in numbers of invading cells was significantly re-increased by pretreatment with 50 μM of pervanadate (Fig. 3c). The 3-D spheroid cell invasion assay showed a reversal of ATO effect, e.g., the protrusion of AGS cells from the spheroid, which was abolished by 10 μM ATO treatment for 24 h, protruded out of the spheroid by 50 μM pervanadate pretreatment (Fig. 3d). Western blot showed that 10 μM ATO treatment induced SHP-1 expression and downregulated p-JAK2/p-STAT3 expression, which in turn, downregulated Snail1 and upregulated E-cadherin expression. These effects were reversed by pretreatment with 50 μM of pervanadate (Fig. 3e). Taken together, these data suggest that SHP-1 may be a key mediator for dephosphorylation of JAK2/STAT3 and inhibition of EMT by ATO treatment in AGS cells.

**Fig. 4:**
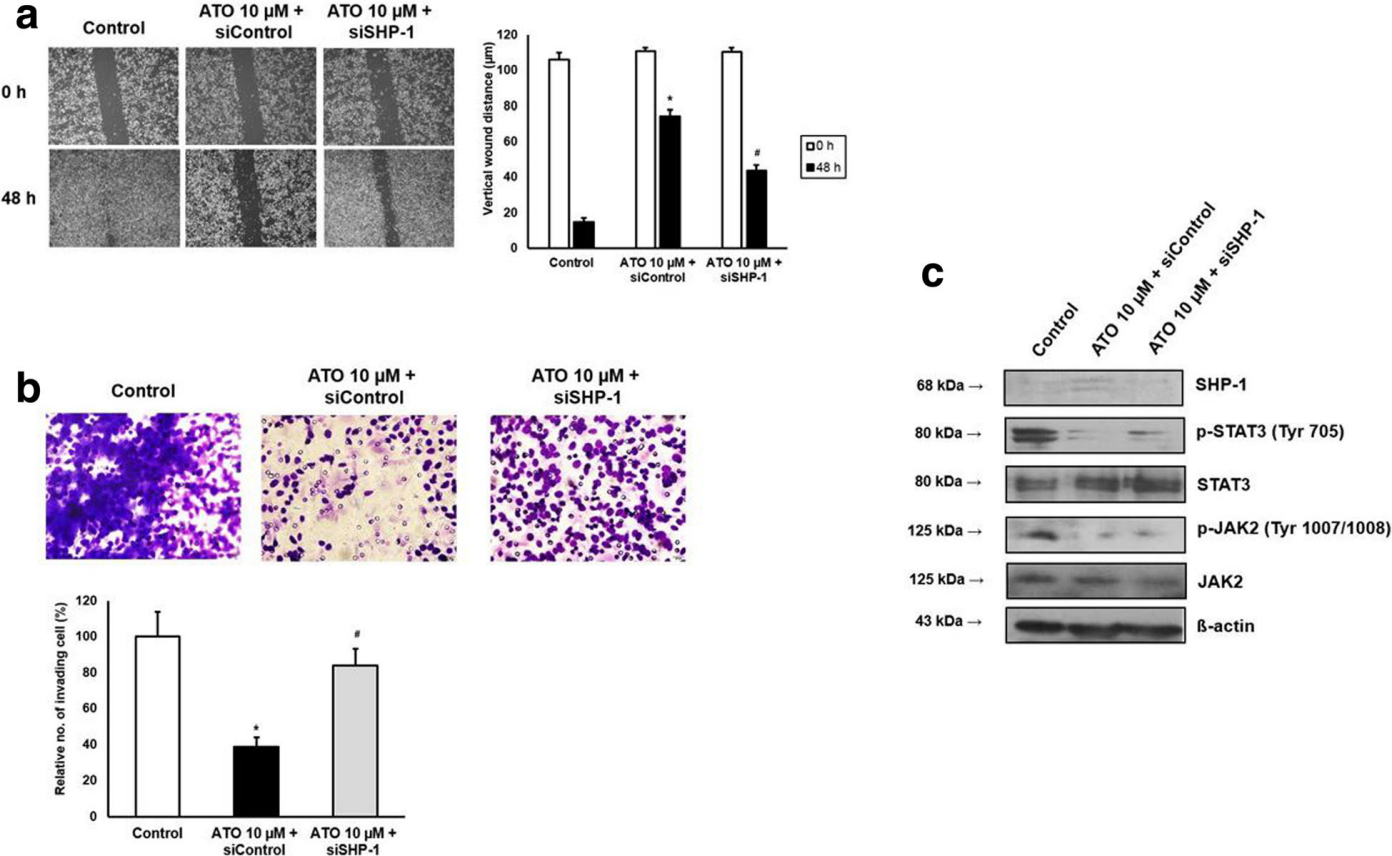
Effects of ATO on AGS cells were reversed by tranfection with SHP-1 siRNA. **a**. Wound closure assay. Left panel; representative images of wound closure. Right panel; analysis of vertical wound distance. Data are presented as mean ± standard deviation. All experiments were performed in triplicate. ^*^P < 0.05, compared with control; ^#^P < 0.05, compared with siControl + ATO 10 μM (n = 3). **b.** Matrigel invasion assay. Left panel; representative images of Matrigel invasion assay. Right panel; analysis of invading cells. The number of positive invading cells was counted under × 20 magnification. Data are presented as mean ± standard deviation. Cell counting was performed in at least 3 randomly selected separate areas. ^*^P < 0.05, compared with control; ^#^P < 0.05, compared with siControl + ATO 10 μM (n = 3). **c**. Western blotting. Whole cell lysate protein was extracted after transfection and ATO treatment. β-actin was used as an internal loading control

To validate the role of SHP-1 on inhibition of STAT-3 activity more specifically, we transfected AGS cells with control or SHP-1 siRNA, then treated with 10 μM ATO for 24 h. siControl-transfected, ATO-treated AGS cells significantly inhibited wound healing process (Fig. 4a) and decreased number of invasive cells (Fig. 4b) compared with naïve AGS cells, however, these effects were reversed by transfection with siSHP-1. Western blotting showed that transfection with siControl followed by ATO treatment upregulated SHP-1 expression and downregulated p-STAT3 and p-JAK2 compared with naïve AGS cells, however, these phenomena were also halved by siSHP-1 (Fig. 4c). The data were consistent with the results from pervanadate-pretreated AGS cells in Fig. 3.

### Anti-tumor effect of ATO is mediated by SHP-1

**Fig. 5:**
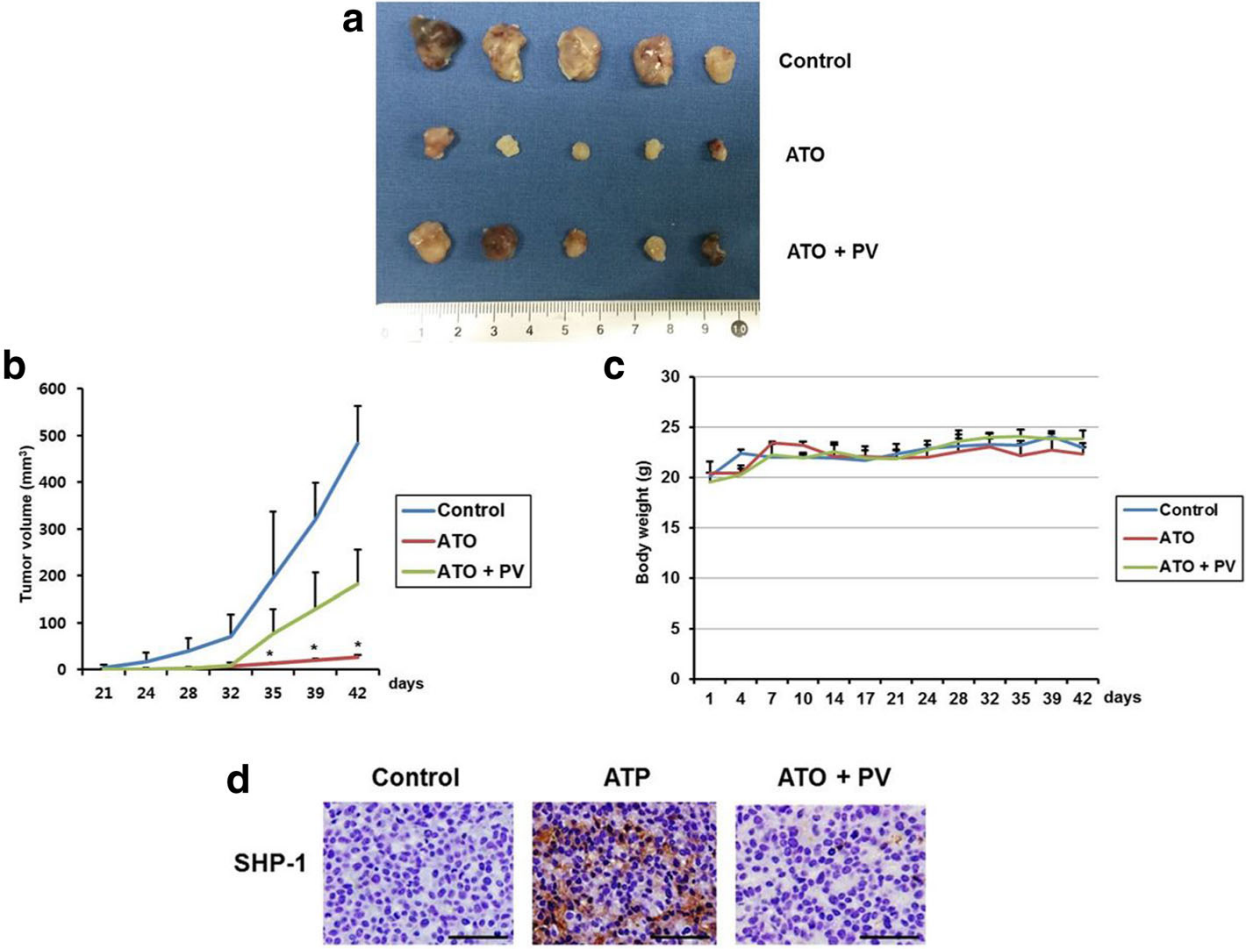
Effects of ATO on xenograft tumor model** a**. Gross tumor extracted from nude mouse after 6 weeks of intraperitoneal administration of ATO with or without pervanadate. **b**. Difference in mean tumor volume among three groups. Data are presented as mean ± standard deviation. ^*^P < 0.05, compared with control (n = 5). **c**. Changes of mean body weight. Data are presented as mean ± standard deviation. **d**. Immunohistochemistry of xenograft tumor. The SHP-1 protein expressions were stained dark brown

The xenograft tumor model showed that intraperitoneal administration of ATO significantly reduced the tumor size and volume compared with those of the control group. However, co-administration of pervanadate with ATO led to a significant increase in tumor volume compared to the control group. There was no significant difference of body weight between the three groups (Fig. 5a-c). By immunohistochemistry stain, tumors from ATO treated mice showed an enhanced staining for SHP-1 in the cytoplasm compared to the controls which were negative for SHP-1. However, co-administration of pervanadate with ATO dramatically reduced the staining for SHP-1 (Fig. 5d). These results suggest that anti-tumorigenic effect of ATO in xenograft in vivo tumor model may be mediated by the induction of SHP-1.

## Discussion

In this study, we showed that ATO inhibits the JAK2/STAT3 signaling axis and EMT process in AGS cells, and that SHP-1 is a key modulator of dephosphorylation of JAK2/STAT3. Inhibition of SHP-1 activity reversed the aforementioned effects of ATO. Furthermore, we showed, in a xenograft tumor model, that the anti-tumor effect of ATO may be mediated by induction of SHP-1 which is also reversed by pharmacologic inhibition of SHP-1. To our knowledge, this is the first study to demonstrate the anti-EMT effect of ATO and its underlying mechanism in gastric cancer cells.

Indeed, STAT3 is a key transcription factor for carcinogenesis, invasion and modulation of microenvironments in gastric cancer. Constitutive activation of STAT3 in gastric epithelial cells promotes cellular proliferation, invasion, and angiogenesis, and inhibits apoptosis by activating various associated target genes including cyclin D1, VEGF-1, Bcl-xL, survivin and MMP-9 [15, 16]., A recent meta-analysis showed that increased p-STAT3 expression in gastric cancer tissue is significantly associated with undifferentiated type, lymph node metastasis and poor prognosis [17]. Furthermore, STAT3 is a crucial mediator in the generation of a cancer-favorable microenvironment in response to cytotoxin-associated antigen (CagA)-positive *Helicobacter pylori* (*H. pylori*) infection in the stomach. For example, once CagA is introduced into gastric epithelial cells, p-STAT3 is activated by stimulation with interleukin-11 (IL-11), and in turn, upregulates various target genes including MMP-7 and CD44v6 to promote cellular invasion [18]. In the surrounding stromal cells, CagA-positive *H. pylori* infection stimulates dendritic cells to produce IL-23, which in turn, activates p-STAT3 in naïve CD4^+^ T-cells and transdifferentiates into T-helper (Th)-17 specific cells [19]. Th-17 predominant cells contribute to the formation of premalignant lesions as well as depth of tumor, lymphovascular invasion and lymph node involvement of gastric cancer [20, 21]. Thus, inhibition of the JAK2/STAT3 signaling pathway is a crucial step for repression of invasion and EMT in gastric cancer.

However, most of the STAT3 inhibitors for gastric cancer have been confined to experimental studies. Several previous in vitro/in vivo studies showed inhibition of STAT3 activity and anti-inflammatory cytokines such as IL-11, IL-6 and IL-1β via repression of JAK1/2 phosphorylation [22, 23]. Several drugs such as AZD 1480, a potent JAK1/2 phosphorylation inhibitor, or OPB-31121, a STAT3 inhibitor targeting Src homology 2 (SH2) domain of STAT3, have been evaluated in phase I and II clinical studies. However, these have been abandoned due to severe side effects [24] or showed relatively low clinical efficacy [25]. Furthermore, technical problems in developing more stable and effective STAT3 inhibitors also exist. Recently, Chen and his colleagues demonstrated that EMT and progression of hepatocellular carcinoma (HCC) is effectively modulated by SHP-1 via suppression of TGF-β1-induced constitutive p-STAT3 activity [26]. In gastric cancer, the role of SHP-1 for inhibition of STAT3 has been rarely reported. Sun et al. showed that transmembrane protein with epidermal growth factor and two follistatin motifs 2 (TMEFF2) is a key binding partner of SHP-1 and effectively suppresses STAT3 signaling in gastric cancer cells. Higher co-expression of TMEFF2 and SHP-1 is closely associated with favorable outcomes in gastric cancer patients [27]. Thus, SHP-1 may be a promising phosphatase for inactivation of STAT3 signaling in gastric cancer cells, and the biologic function of SHP-1 in gastric cancer needs to be further evaluated in future studies.

We previously reported that expression of SHP-1 is mostly attenuated or abolished in gastric cancer cell lines which are governed by epigenetic mechanism [8]. Sun et al. showed that mRNA expression of SHP-1 is highest in normal gastric tissues, followed by intestinal metaplasia, dysplasia, and lowest in gastric cancer. Inversely, CpG island promoter hypermethylation of SHP-1 was most frequent in gastric cancer, followed by dysplasia, intestinal metaplasia and normal gastric tissues [27]. Thus, upregulation of SHP-1 might be an important issue for effective dephosphorylation and inhibition of STAT3 in gastric cancer cells. Chen et al. showed that several multikinase inhibitors including sorafenib, dovitinib and regorafenib, and their analogues, have potent anti-STAT3 effects via induction of SHP-1 in HCC cells [28–31]. In gastric cancer, Liu et al. experimentally showed that honokiol, a small-molecular weight natural product, increases SHP-1 expression and dephosphorylates STAT-3 through upregulation of calpain II, a calcium-activated non-lysosomal cysteine protease, in gastric cancer cells [32]. In this study, we showed that ATO enhances SHP-1 expression to dephosphorylate JAK2/STAT3 and modulate Snail1/E-cadherin expression, thus inhibiting EMT and cellular invasion in AGS cells. Therefore, we consider that SHP-1 is a key regulator of dephosphorylation of STAT3 and has an anti-EMT effect in gastric cancer cells. Meanwhile, a recent study showed that ATO shows a reductive effect on multidrug resistance to doxorubicin in gastric cancer cell lines [33]. Constitutive activation of STAT3 is closely associated with chemoresistance in gastric cancer [34]. Restoration of chemoresistance by ATO in gastric cancer cells might be related to inactivation of STAT3 via induction of SHP-1. This needs to be investigated further.

## Conclusion

ATO effectively inhibits cellular invasion, EMT, and tumorigenesis in gastric cancer cells which are mediated by dephosphorylation of JAK2/STAT3 through increase of SHP-1 expression. Since direct inhibition of STAT3 is technically difficult and is limited in clinical efficacy, modulation of the SHP-1/STAT3 axis might be a promising therapeutic strategy in the treatment of gastric cancer, especially in overcoming chemoresistance. Further research for the development of potent and stable SHP-1 inducers is expected in the near future.

## Abbreviations

ATO: Arsenic trioxide
CagA: Cytotoxin-associated antigen
EMT: Epithelial-mesenchymal transition
*H. pylori*: *Helicobacter pylori*
HCC: Hepatocellular carcinoma
IHC: Immunohistochemistry
IL: Interleukin
JAK2: Janus kinase 2
MMP-9: Matrix metalloproteinases-9
PI3K: Phosphatidylinositol 3-kinase
SH2: Src homology 2
SHP-1: SH2- containing protein tyrosine phosphatase 1
STAT3: Signal transducer and activator of transcription 3
Th: T-helper
TMEFF2: Transmembrane protein with epidermal growth factor and two follistatin motifs 2
VEGF-1: Vascular endothelial growth factor-1
WST-1: Water-soluble tetrazolium salt-1

## References

[1] Li Y, Qu X, Qu J, Zhang Y, Liu J, Teng Y, Hu X, Hou K, Liu Y (2009). Arsenic trioxide induces apoptosis and G2/M phase arrest by inducing Cbl to inhibit PI3K/Akt signaling and thereby regulate p53 activation. Cancer Lett.

[2] Gao YH, Zhang HP, Yang SM, Yang Y, Ma YY, Zhang XY, Yang YM (2014). Inactivation of Akt by arsenic trioxide induces cell death via mitochondrial-mediated apoptotic signaling in SGC-7901 human gastric cancer cells. Oncol Rep.

[3] Jia Y, Liu D, Xiao D, Ma X, Han S, Zheng Y, Sun S, Zhang M, Gao H, Cui X (2013). Expression of AFP and STAT3 is involved in arsenic trioxide-induced apoptosis and inhibition of proliferation in AFP-producing gastric cancer cells. PLoS One.

[4] Lamouille S, Xu J, Derynck R (2014). Molecular mechanisms of epithelial-mesenchymal transition. Nat Rev Mol Cell Biol.

[5] Yue P, Turkson J (2009). Targeting STAT3 in cancer: how successful are we?. Expert Opin Investig Drugs.

[6] Lopez-Ruiz P, Rodriguez-Ubreva J, Cariaga AE, Cortes MA, Colas B (2011). SHP-1 in cell-cycle regulation. Anti Cancer Agents Med Chem.

[7] Zhang J, Somani AK, Siminovitch KA (2000). Roles of the SHP-1 tyrosine phosphatase in the negative regulation of cell signalling. Semin Immunol.

[8] Joo MK, Park JJ, Yoo HS, Lee BJ, Chun HJ, Lee SW, Bak YT (2016). Epigenetic regulation and anti-tumorigenic effects of SH2-containing protein tyrosine phosphatase 1 (SHP1) in human gastric cancer cells. Tumour biology : the journal of the International Society for Oncodevelopmental Biology and Medicine.

[9] Huang Z, Lee H, Lee E, Kang SK, Nam JM, Lee M (2011). Responsive nematic gels from the self-assembly of aqueous nanofibres. Nat Commun.

[10] Liao WT, Jiang D, Yuan J, Cui YM, Shi XW, Chen CM, Bian XW, Deng YJ, Ding YQ (2011). HOXB7 as a prognostic factor and mediator of colorectal cancer progression. Clinical cancer research : an official journal of the American Association for Cancer Research.

[11] Petit V, Massonnet G, Maciorowski Z, Touhami J, Thuleau A, Nemati F, Laval J, Chateau-Joubert S, Servely JL, Vallerand D (2013). Optimization of tumor xenograft dissociation for the profiling of cell surface markers and nutrient transporters. Laboratory investigation; a journal of technical methods and pathology.

[12] Kaufhold S, Bonavida B (2014). Central role of Snail1 in the regulation of EMT and resistance in cancer: a target for therapeutic intervention. Journal of experimental & clinical cancer research : CR.

[13] Lee JH, Chiang SY, Nam D, Chung WS, Lee J, Na YS, Sethi G, Ahn KS (2014). Capillarisin inhibits constitutive and inducible STAT3 activation through induction of SHP-1 and SHP-2 tyrosine phosphatases. Cancer Lett.

[14] Pandey MK, Sung B, Aggarwal BB (2010). Betulinic acid suppresses STAT3 activation pathway through induction of protein tyrosine phosphatase SHP-1 in human multiple myeloma cells. Int J Cancer.

[15] Jackson CB, Giraud AS (2009). STAT3 as a prognostic marker in human gastric cancer. J Gastroenterol Hepatol.

[16] Joo MK, Park JJ, Kim SH, Yoo HS, Lee BJ, Chun HJ, Lee SW, Bak YT (2015). Antitumorigenic effect of plumbagin by induction of SH2-containing protein tyrosine phosphatase 1 in human gastric cancer cells. Int J Oncol.

[17] Ji K, Zhang L, Zhang M, Chu Q, Li X, Wang W (2016). Prognostic value and Clinicopathological significance of p-stat3 among gastric carcinoma patients: a systematic review and meta-analysis. Medicine.

[18] Han JC, Zhang KL, Chen XY, Jiang HF, Kong QY, Sun Y, Wu ML, Huang L, Li H, Liu J (2007). Expression of seven gastric cancer-associated genes and its relevance for Wnt, NF-kappaB and Stat3 signaling. APMIS : acta pathologica, microbiologica, et immunologica Scandinavica.

[19] Giraud AS, Menheniott TR, Judd LM (2012). Targeting STAT3 in gastric cancer. Expert Opin Ther Targets.

[20] Iida T, Iwahashi M, Katsuda M, Ishida K, Nakamori M, Nakamura M, Naka T, Ojima T, Ueda K, Hayata K (2011). Tumor-infiltrating CD4+ Th17 cells produce IL-17 in tumor microenvironment and promote tumor progression in human gastric cancer. Oncol Rep.

[21] Liu X, Jin H, Zhang G, Lin X, Chen C, Sun J, Zhang Y, Zhang Q, Yu J (2014). Intratumor IL-17-positive mast cells are the major source of the IL-17 that is predictive of survival in gastric cancer patients. PLoS One.

[22] Stuart E, Buchert M, Putoczki T, Thiem S, Farid R, Elzer J, Huszar D, Waring PM, Phesse TJ, Ernst M (2014). Therapeutic inhibition of Jak activity inhibits progression of gastrointestinal tumors in mice. Mol Cancer Ther.

[23] Judd LM, Menheniott TR, Ling H, Jackson CB, Howlett M, Kalantzis A, Priebe W, Giraud AS (2014). Inhibition of the JAK2/STAT3 pathway reduces gastric cancer growth in vitro and in vivo. PLoS One.

[24] Buchert M, Burns CJ, Ernst M (2016). Targeting JAK kinase in solid tumors: emerging opportunities and challenges. Oncogene.

[25] Oh DY, Lee SH, Han SW, Kim MJ, Kim TM, Kim TY, Heo DS, Yuasa M, Yanagihara Y, Bang YJ (2015). Phase I study of OPB-31121, an oral STAT3 inhibitor, in patients with advanced solid tumors. Cancer research and treatment : official journal of Korean Cancer Association.

[26] Fan LC, Shiau CW, Tai WT, Hung MH, Chu PY, Hsieh FS, Lin H, Yu HC, Chen KF (2015). SHP-1 is a negative regulator of epithelial-mesenchymal transition in hepatocellular carcinoma. Oncogene.

[27] Sun T, Du W, Xiong H, Yu Y, Weng Y, Ren L, Zhao H, Wang Y, Chen Y, Xu J (2014). TMEFF2 deregulation contributes to gastric carcinogenesis and indicates poor survival outcome. Clinical cancer research : an official journal of the American Association for Cancer Research.

[28] Chen KF, Tai WT, Liu TH, Huang HP, Lin YC, Shiau CW, Li PK, Chen PJ, Cheng AL (2010). Sorafenib overcomes TRAIL resistance of hepatocellular carcinoma cells through the inhibition of STAT3. Clinical cancer research : an official journal of the American Association for Cancer Research.

[29] Tai WT, Cheng AL, Shiau CW, Liu CY, Ko CH, Lin MW, Chen PJ, Chen KF (2012). Dovitinib induces apoptosis and overcomes sorafenib resistance in hepatocellular carcinoma through SHP-1-mediated inhibition of STAT3. Mol Cancer Ther.

[30] Tai WT, Chu PY, Shiau CW, Chen YL, Li YS, Hung MH, Chen LJ, Chen PL, Su JC, Lin PY (2014). STAT3 mediates regorafenib-induced apoptosis in hepatocellular carcinoma. Clinical cancer research : an official journal of the American Association for Cancer Research.

[31] Tai WT, Shiau CW, Chen PJ, Chu PY, Huang HP, Liu CY, Huang JW, Chen KF (2014). Discovery of novel Src homology region 2 domain-containing phosphatase 1 agonists from sorafenib for the treatment of hepatocellular carcinoma. Hepatology.

[32] Liu SH, Wang KB, Lan KH, Lee WJ, Pan HC, Wu SM, Peng YC, Chen YC, Shen CC, Cheng HC (2012). Calpain/SHP-1 interaction by honokiol dampening peritoneal dissemination of gastric cancer in nu/nu mice. PLoS One.

[33] Zhao YY, Yu L, Liu BL, He XJ, Zhang BY (2015). Downregulation of P-gp, Ras and p-ERK1/2 contributes to the arsenic trioxide-induced reduction in drug resistance towards doxorubicin in gastric cancer cell lines. Mol Med Rep.

[34] Huang S, Chen M, Ding X, Zhang X, Zou X (2013). Proton pump inhibitor selectively suppresses proliferation and restores the chemosensitivity of gastric cancer cells by inhibiting STAT3 signaling pathway. Int Immunopharmacol.

